# Emerging strengths in Asia Pacific bioinformatics

**DOI:** 10.1186/1471-2105-9-S12-S1

**Published:** 2008-12-12

**Authors:** Shoba Ranganathan, Wen-Lian Hsu, Ueng-Cheng Yang, Tin Wee Tan

**Affiliations:** 1 Department of Chemistry and Biomolecular Sciences and ARC Centre of Excellence in Bioinformatics, Macquarie University, Sydney NSW 2109, Australia; 2 Department of Biochemistry, Yong Loo Lin School of Medicine, National University of Singapore, 8 Medical Drive, Singapore 117597; 3 Institute of Information Science, Academia Sinica, Nankang, Taipei, Taiwan, ROC; 4 Department of Computer Science, National Tsing-Hua University, Hsinchu, Taiwan, ROC; 5 Institute of Biomedical Informatics and Center for Systems and Synthetic Biology, National Yang-Ming University, Taiwan, ROC

## Abstract

The 2008 annual conference of the Asia Pacific Bioinformatics Network (APBioNet), Asia's oldest bioinformatics organisation set up in 1998, was organized as the 7^th ^International Conference on Bioinformatics (InCoB), jointly with the Bioinformatics and Systems Biology in Taiwan (BIT 2008) Conference, Oct. 20–23, 2008 at Taipei, Taiwan. Besides bringing together scientists from the field of bioinformatics in this region, InCoB is actively involving researchers from the area of systems biology, to facilitate greater synergy between these two groups. Marking the 10^th ^Anniversary of APBioNet, this InCoB 2008 meeting followed on from a series of successful annual events in Bangkok (Thailand), Penang (Malaysia), Auckland (New Zealand), Busan (South Korea), New Delhi (India) and Hong Kong. Additionally, tutorials and the Workshop on Education in Bioinformatics and Computational Biology (WEBCB) immediately prior to the 20th Federation of Asian and Oceanian Biochemists and Molecular Biologists (FAOBMB) Taipei Conference provided ample opportunity for inducting mainstream biochemists and molecular biologists from the region into a greater level of awareness of the importance of bioinformatics in their craft. In this editorial, we provide a brief overview of the peer-reviewed manuscripts accepted for publication herein, grouped into thematic areas. As the regional research expertise in bioinformatics matures, the papers fall into thematic areas, illustrating the specific contributions made by APBioNet to global bioinformatics efforts.

## Historical notes

Established in 1998 [1], the Asia-Pacific Bioinformatics Network (APBioNet, [2-4]) strives to bring together scientists from diverse disciplines, working together to advance the frontiers of bioinformatics. Following annual meetings held at the Pacific Symposium of Biocomputing (1998–2001), APBioNet executive committee members assisted in organizing InCoB2002 (the International Conference on Bioinformatics, 2002) in Bangkok and adopted this meeting as their annual conference. Since then, InCoB meetings have been held in Penang, Malaysia (2003); Auckland, New Zealand (2004); Busan, South Korea (2005), New Delhi, India (2006) and Hong Kong/Hanoi (by videocasting) (2007), with InCoB2008 at Taipei, Taiwan [5], with attendance rising to the highest ever (over 1,000) in Delhi.

Pursuant to APBionet's initial efforts of the BioMirrors initiative, in collaboration with the Asia Pacific Advanced Network (APAN), [6] to develop the network infrastructure for maintaining bioinformatics databases and computational resources, Sangket *et al*. [7] developed a BitTorrent-based Peer-to-Peer (btP2P) file distribution model for automatic synchronization and distribution of large amounts of biological data suitable for developing countries which are connected with developed countries via the TransEurasia Information Network TEIN2 initiative. To facilitate replication of bioinformatics resource nodes, APBioNet was awarded a grant by the International Development Research Centre of Canada (IDRC) to build a user-friendly package APBioBox (2003) with Globus grid software for fast deployment of grid computing for bioinformatics applications and subsequently collaborated with Sun Microsystems' Asia Pacific Science and Technology Center (APSTC) to co-develop the BioCluster Grid on the Solaris platform. It facilitated the development of a Knoppix LiveCD of bioinformatics applications called APBioKnoppix (2004), and subsequently in conjunction with the ASEAN SubCommittee on Biotechnology and the National University of Singapore's Bioinformatics Centre, the BioSlax Unix bioinformatics package of LiveCD and bioinformatics on a thumb drive (2006) was developed. More recently, this was extended to a VMware virtual machine version (for Windows and Mac) and a BioSlax Server Version for rapid (5 min) deployment of a complete bioinformatics computational resource for a teaching or research laboratory with a 1 Terabyte USB external hard disk of BioMirrors and BLAST datasets (see ), test-deployed in Vietnam and elsewhere in 2007. These activities represent APBioNet's growing efforts at contributing to the software development scene for bioinformatics, instead of being solely a passive recipient of technology.

While constantly striving to establish better access to data and analysis tools, APBioNet's priorities also extended to education and training of the life science community, with active participation in initiatives such as the S* Life Science Informatics Alliance [8] and Workshops on Education in Bioinformatics (WEB, initiated by SR) [9], to meld bioinformatics with mainstream life science research. Outreach training workshops and courses expanded to diverse locations in Asia were facilitated by sponsorships from FAOBMB, IUBMB and UNESCO, and this has resulted in a much greater awareness and interest amongst regional scientists in the various aspects of bioinformatics. In recent years the Asia Pacific region has shown the fastest growth in bioinformatics publications (D. Seaton, Elsevier, personal communication), and has hosted several reputable international conferences, with the past year alone supporting the Genome Informatics Workshop (GIW) in Singapore [10], the Asia Pacific Bioinformatics Conference (APBC) in Hong Kong and the Research in Computational Molecular Biology (RECOMB) Conference also in Singapore [11], culminating in InCoB2008 in Taipei. This trend perhaps foreshadows the location of the largest bioinformatics global meeting, the Intelligent Systems in Molecular Biology (ISMB) conference of the International Society for Computational Biology [12] in the Asia Pacific region in the near future.

Having reached an international peer-reviewed high-impact factor standard in journal publication, APBioNet embarked on further raising the standards for the region by publishing a dedicated *BMC Bioinformatics *supplement, starting in 2006 [13,14]. By 2008, manuscripts from APBioNet members have diversified and increased in quality and sophistication, and are now focussed on emerging bioinformatics research areas, including microRNAs; transcriptomes, SNPs and protein analyses as well as protein trafficking, systems biology, immunoinformatics, and biodiversity informatics, further considered below.

## Review policy

Papers submitted to these proceedings were peer-reviewed by at least two reviewers, from the APBioNet/InCoB program committee members and external experts as required (listed in Additional File 1). InCoB2008 also provided multi-track submissions, with the inclusion of new sessions to present research highlights from recent publications and for showcasing technology developments. With tutorials aimed at introducing new concepts and advancing current practices, and a specialist workshop on bioinformatics education, InCoB2008 has achieved the status of a comprehensive international bioinformatics meeting in the Asia Pacific.

The aim of the editors was to select only the best papers from more than a dozen Asia Pacific countries. From the 47 full paper submissions, 33 were short listed for oral presentation. This supplement features 24 papers, reflecting an overall acceptance rate of 51%, with seven more appearing in the online journal, *Bioinformation *[15]. The impressive extent of collaboration in bioinformatics research in the region is demonstrated by the permutations of co-authorship, variously involving papers from Australia, China, Hong Kong, India, Japan, Korea, Singapore, Taiwan, Thailand, UK and USA. A brief review of the various themes follows.

## MicroRNAs

The discovery in 2004 of very short regulatory RNA segments, termed micro RNAs (miRNAs), has provoked intense research. Chang *et al. *[16] propose a novel relaxed variable kernel density estimator for predicting species-specific miRNA precursors, particularly suited to taxa distant from humans. The identification of false positives from such predictions is provided by Leung *et al. *[17], using a clustering approach. The biomolecules that these miRNAs bind to are predicted accurately by the support vector machine classification method of Yang *et al. *[18] while Tran *et al. *[19] explore the regulatory networks controlled by miRNAs.

## Transcriptome analysis

The regulatory role of RNAs is dependent on their binding to specific proteins. Cheng *et al. *[20] present a novel position-specific scoring matrix method, of high sensitivity, for predicting the RNA-binding sites on proteins. Transcription regulation depends on the binding of several factors based on the presence of distinct signal regions. Lu *et al. *[21] propose a new algorithm to extract these transcription factor binding sites from a set of unaligned gene sequences. Eukaryotic genes are assembled from a set of exons, from which the intervening intronic elements have been spliced out. A new feature-based splice site detection method has been developed by Baten *et al. *[22] to accurately delineate the architecture of a multi-exonic gene.

## SNPs

Single nucleotide polymorphisms (SNPs) are key elements in genomics research, importantly for correlating genetic variations with disease outcomes. VarDetect (Ngamphiw *et al. *[23]) is a new generation tool for the accurate detection of SNPs, while the functional implications of these variations can be assessed using FANS (Liu *et al. *[24]).

## Protein analysis

Understanding protein interactions and networks can provide a deep insight into the functions of biological molecules generally. Lee *et al. *[25] have extended our knowledge of protein interactions to 18 species, using orthologous interacting pairs. Interactions between proteins are characterized by the surface features of their three dimensional structures. Chang *et al. *[26] propose a new approach to compute protein solvent accessibility, which will facilitate protein tertiary structure prediction from sequence. The conservation of functional residues in a protein or a domain is critical to maintaining biological function. The evolutionary forces acting on mutation sites can be appraised with WPSMaker (Lee *et al. *[27]). The functional significance of active site residues in an enzyme has been explored by *in silico *ligand docking by Gowthaman *et al. *[28], in the interests of developing anti-HIV drugs.

## Protein trafficking

Proteins are functional only if they are transported to specific cellular compartments. Choo and Ranganathan [29] report the involvement of flanking residues from the mature protein moiety in a large-scale analysis of experimentally validation secretory signal peptides, while Mizuno *et al. *[30] have experimentally evaluated predicted peroxisomal targeting signals.

## Systems biology

Temporal changes in the interactions and concentrations of biological molecules are critical to systems biology. Using model systems, Wu *et al. *[31] present a new approach to dynamic sensitivity analysis.

## Immunoinformatics

As part of our quest to efficiently address healthcare issues, delving through the ever-increasing biomedical literature presents an immense challenge. Tsai *et al *[32] propose a new system to convert their machine-learning based text analysis output into standardized biomedical annotations. Yang *et al. *[33] have compiled a database linking genetic variations to disease, while Wang *et al. *[34] address the issue of immunoglobulin heavy chain diversity to allow understanding antibody maturation and antibody-based therapy design. Lim *et al. *[35] propose a new method for predicting the allergenicity of a protein from its sequence information while Lin *et al. *[36] have evaluated the efficacy of available major histocompatibility complex Class II peptide binding prediction servers for effective vaccine design. Hsu *et al. *[37] have combined candidate disease gene information for genetic studies with protein interaction networks to propose a pathogenetic disease model for schizophrenia.

## Biodiversity informatics

The application of bioinformatics approaches to data from several other areas such as biodiversity and ecology is only logical [9]. In this area, Lim *et al. *[38] have developed an integrated Korean biodiversity and genetic information retrieval system to link species information with molecular data. Gaikwad *et al. *[39] have created a customary medicinal plant knowledge base, impressively integrating taxonomy, phytochemistry, biogeography and the biological activities of customary medicinal plant species with chemoinformatics, to facilitate the discovery of novel bioactive compounds.

## Conclusion

Considering the scope and depth of topics covered in this issue, Asia Pacific bioinformatics research has maintained its level of research achievement, previously set with high standards in 2006. This area is now acknowledged to be a core research discipline in our educational and research institutions. The efforts expended into setting up the bioinformatics resource infrastructure have started to yield dividends, with a number of papers bearing the names of graduate research students as first authors.

Further, bioinformatics is now considered essential to provide substantial support to genomics and health research, as reported by workers from Thailand [40]. Regional meetings such as the recent Second ASEAN-China International Bioinformatics Workshop and the Third East Asia Bioinformation Network meeting [41], held in Singapore in 2008, focus on specific applications of local relevance and cooperation, such as bioinformatics in traditional medicines and in emerging infectious diseases. Such endeavours are essential in addressing issues on human resource development in bioinformatics and in combating global health threats. After 10 years, APBioNet is well positioned "to foster the growth of Bioinformatics and its allied disciplines in the Asia Pacific" [2], and to sustain a new generation of researchers in the 'post-omic' era.

## Competing interests

The authors declare that they have no competing interests.

## Supplementary Material

Additional File 1 APBioNet InCoB2008 Program Committee members. Click here for file
