# Supplementary material for: Emerging strengths in Asia Pacific bioinformatics
Source: BMC Bioinformatics. 2008 Dec 12;9(Suppl 12):S1. doi: 10.1186/1471-2105-9-S12-S1 (PMC2638166; doi:10.1186/1471-2105-9-S12-S1)
Supplement: Additional File 1 — APBioNet InCoB2008 Program Committee members. [file 1471-2105-9-S12-S1-S1.pdf]

Emerging strengths in Asia Pacific bioinformatics

by Shoba Ranganathan, Wen-Lian Hsu, Ueng-Cheng Yang and Tin-Wee Tan

Additional File 1: APBioNet InCoB2008 Program committee members

1. Jonathan **Arthur**, University of Sydney, Australia
2. Vladimir **Bajic**, South African National Bioinformatics Institute, S. Africa
3. Christopher **Baker**, Institute for Infocomm Research, Singapore
4. Alex **Bateman**, The Wellcome Trust Sanger Institute, UK
5. Jong **Bhak**, Korean Bio Information Center (KOBIC), Korea
6. Vladimir **Brusic,** Dana Faber Cancer Institute, USA
7. Michael **Charleston**, University of Sydney, Australia
8. Vijayalakshmi **Chelliah**, European Bioinformatics Institute, UK
9. Xin **Chen**, Zhejiang University, China
10. Khar Heng **Choo**, Institute for Infocomm Research, Singapore
11. Adrian **Cootes**, Macquarie University, Australia
12. Terry **Gaasterland**, University of California, San Diego, USA
13. Jitendra **Gaikwad**, Macquarie University, Australia
14. Chia-Lang **Hsu**, National Yang Ming University, Taiwan
15. Ming-Jing **Hwang**, Academia Sinica, Taiwan
16. Mohammad **Islam**, Macquarie University, Australia
17. Asif **Khan**, National University of Singapore, Singapore
18. Javed **Khan**, Macquarie University, Australia
19. Varun **Khanna**, Macquarie University, Australia
20. Judith **Klein-Seetharaman**, University of Pittsburgh School of Medicine, USA
21. Gaurav **Kumar**, Macquarie University, Australia
22. Michael A. **Langston**, University of Tennessee, USA
23. Jingchu **Luo**, Peking University, China
24. Ranjeeta **Menon**, Macquarie University, Australia
25. Olivo **Miotto**, University of Oxford, UK
26. Satoru **Miyano**, University of Tokyo, Japan
27. Shivashankar **Nagaraj**, Macquarie University, Australia
28. H.A. **Nagarajaram**, Center for DNA Fingerprinting and Diagnostics (CDFD), India
29. Kenta **Nakai**, University of Tokyo, Japan
30. Haruki **Nakamura**, Osaka University, Japan
31. Wailap Victor **Ng**, National Yang Ming University, Taiwan
32. Mohd Shahir Shamsir **Omar**, University Teknologi Malaysia (UTM), Malaysia
33. Ian **Paulsen**, Macquarie University, Australia
34. Meena **Sakharkar**, Nanyang Technological University, Singapore
35. Christian **Schönbach**, Nanyang Technological University, Singapore
36. Amandeep **Sidhu**, Curtin University of Technology, Australia
37. Daniel **Sze**, Hong Kong Polytechnic University, Hong Kong
38. Martti **Tammi,** National University of Singapore, Singapore
39. Rohan **Teasdale**, University of Queensland, Australia
40. Joo Chuan **Tong**, Institute for Infocomm Research, Singapore
41. Chandra **Verma**, Bioinformatics Institute, Singapore
42. Lawrence **Wee**, National University of Singapore, Singapore
43. Limsoon **Wong**, National University of Singapore, Singapore
44. Guang Lan **Zhang**, Dana Farber Cancer Institute, USA
45. Louxin **Zhang**, National University of Singapore, Singapore
46. Albert **Zomaya**, University of Sydney, Australia
47. Michael **Zuker**, Rensselaer Polytechnic Institute, USA
